# Ssu72 Dual-Specific Protein Phosphatase: From Gene to Diseases

**DOI:** 10.3390/ijms22073791

**Published:** 2021-04-06

**Authors:** Soeun Hwang, Min-Hee Kim, Chang-Woo Lee

**Affiliations:** 1 Department of Molecular Cell Biology, Samsung Medical Center, Sungkyunkwan University School of Medicine, Suwon 16419, Korea; thdmsdlrh@naver.com (S.H.); juhy031@naver.com (M.-H.K.); 2 SKKU Institute for Convergence, Sungkyunkwan University, Suwon 16419, Korea; 3 Curogen Technology, Suwon 16419, Korea

**Keywords:** Ssu72 phosphatase, gene expression, cell cycle, T cell immunity, metabolism

## Abstract

More than 70% of eukaryotic proteins are regulated by phosphorylation. However, the mechanism of dephosphorylation that counteracts phosphorylation is less studied. Phosphatases are classified into 104 distinct groups based on substrate-specific features and the sequence homologies in their catalytic domains. Among them, dual-specificity phosphatases (DUSPs) that dephosphorylate both phosphoserine/threonine and phosphotyrosine are important for cellular homeostasis. Ssu72 is a newly studied phosphatase with dual specificity that can dephosphorylate both phosphoserine/threonine and phosphotyrosine. It is important for cell-growth signaling, metabolism, and immune activation. Ssu72 was initially identified as a phosphatase for the Ser5 and Ser7 residues of the C-terminal domain of RNA polymerase II. It prefers the *cis* configuration of the serine–proline motif within its substrate and regulates Pin1, different from other phosphatases. It has recently been reported that Ssu72 can regulate sister chromatid cohesion and the separation of duplicated chromosomes during the cell cycle. Furthermore, Ssu72 appears to be involved in the regulation of T cell receptor signaling, telomere regulation, and even hepatocyte homeostasis in response to a variety of stress and damage signals. In this review, we aim to summarize various functions of the Ssu72 phosphatase, their implications in diseases, and potential therapeutic indications.

## 1. Introduction

Phosphorylation and dephosphorylation processes are important for the maintenance of homeostatic balance in molecular and cellular signaling. More than 70% of all cellular proteins are regulated by phosphorylation. According to human genome analyses, 518 kinases are either tyrosine (Tyr) kinases or serine/threonine (Ser/Thr) kinases. The total number of Tyr phosphatases and Ser/Thr phosphatases is approximately 137, many fewer than the number of the abovementioned kinases [1,2]. Thus, protein phosphatases need to be tightly regulated, depending on the specific substrate, particularly in terms of balance with their kinase counterparts, in a wide range of cellular processes. Protein kinases and phosphatases can promote the transfer of phosphate between substrates. One phosphatase can modify a variety of substrate proteins, and one substrate protein can be regulated by several phosphatases. However, some phosphatases can catalyze the dephosphorylation of non-protein substrates [3,4]. DUSPs can dephosphorylate both tyrosine and serine/threonine residues [5].

Ssu72 is a relatively well-elucidated DUSP. It was initially recognized in the yeast *Saccharomyces cerevisiae* with the ability to control transcription initiation [6]. Ssu72 is essential for RNA polymerase II (RNAPII) transcription and the termination of the 3’-end processing of RNA [7]. Interestingly, Ssu72 bears some similarity in its protein structure to a low-molecular-weight phosphotyrosine phosphatase, Ltp1 [8]. However, Ssu72 has a unique active site with specific structural characteristics at the C-terminus. It consists of a central 5-stranded β-sheet (β1–β5) enclosed by helices on both sides. Two-stranded antiparallel β-sheets (β2A and β2B) are close to the active site [9]. In addition, the C-terminus of Ssu72 comprises an extra helix (αG) and β-strand (β5). The αD helix present in Ssu72 can affect phosphopeptide binding [9]. Several recent studies have shown that Ssu72 plays a critical role in various signaling mechanisms as a protein phosphatase in addition to its role in transcriptional regulation. Ssu72 is recruited by fission yeast Pin1 peptidyl-prolyl isomerase in RNAPII to promote carboxyl-terminal domain (CTD) dephosphorylation for transcriptional elongation [10]. In this review, we focus on various functions of Ssu72 and their pathological associations by either loss- or gain-of-function and discuss the potential therapeutic implications.

## 2. Mechanism by Which Ssu72 Phosphatase Regulates Gene Expression

Ssu72 plays an essential role in dephosphorylating phosphorylated Serine 5 (Ser5P) and Serine 7 (Ser7P) of the carboxyl-terminal domain (CTD) of RNAPII [6,8,11]. Ssu72 contains the cysteine-X(5)-arginine (CX5R) signature motif of protein tyrosine phosphatases (PTPases). Mutations of Cys residues can lead to the loss of Ssu72′s phosphatase activity [12]. In addition, aspartate loops in the active site of Ssu72 seem to be essential for maintaining the phosphatase activity of Ssu72.

Ssu72 has a dual function in transcriptional biogenesis by interacting with many kinases and/or phosphatases and multiple transcription factors. It has been reported that Ssu72 can enhance a TFIIB defect in yeast and facilitate the assembly of the transcription preinitiation complex (PIC), which is important for accurate start site selection [13]. Ssu72 influences PIC formation and stability by cooperating with RNAPII subunits and other kinases/phosphatases to regulate transcriptional transition. Additionally, Ssu72 can regulate the 3′-end processing of nascent mRNA and gene looping [14,15,16].

### 2.1. Ssu72 Acts as an RNA Polymerase II CTD Phosphatase in the Transcription Cycle

The CTD of RNA polymerase B1 (Rpb1), the largest subunit of RNAPII, is crucial for the regulation of distinct steps in transcription, which is composed of three main stages (initiation, elongation, and termination) in eukaryotes. The CTD is composed of heptad repeats with the consensus motif Tyr1–Ser2–Pro3–Thr4–Ser5–Pro6–Ser7. The length of its repeat sequence differs between species, ranging from 26 repeats in budding yeast to 52 repeats in mammals. The sequence of the CTD undergoes dynamic structural changes through multiple post-translational modifications, including phosphorylation/dephosphorylation, *cis–trans* Pro isomerization, and *O*-GlcNAcylation [17,18,19,20]. Flexible interactions between the CTD and diverse factors at appropriate stages of the transcription process are critical for the regulation of multiple steps of the transcription cycle. They are also essential for the successful transition of the transcription cycle and transcriptional processing, such as the 3′-end processing of nascent mRNA [21,22].

**Figure 1:**
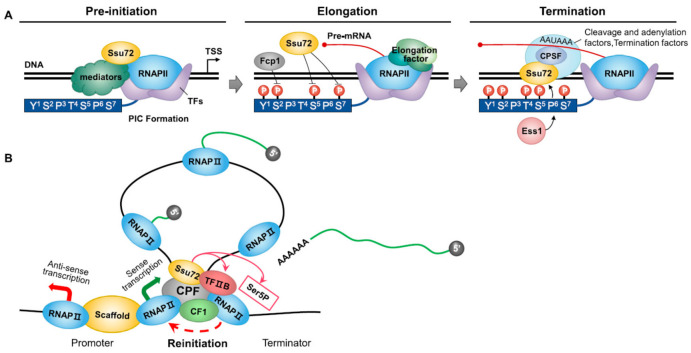
Summary of the functional role of Ssu72 phosphatase in transcription cycle. (**A**) During the initiation of transcription, Ssu72 can genetically and functionally interact with RNAPII subunits, TFIIB, and multiple kinases/phosphatases associated with carboxyl-terminal domain (CTD) modification, contributing to preinitiation complex (PIC) formation and transcription start site selection by RNA polymerase II (RNAPII). Furthermore, Ssu72 can regulate initiation–elongation and elongation–termination transitions by dephosphorylating both Ser5P and Ser7P. For example, Ssu72 along with Ser2P phosphatase Fcp1 can restore the hypophosphorylated form of CTD during elongation and induce Ser5P–Pro6 CTD conformation by interacting with Ess1, a proline isomerase, during transcription termination. (**B**) In the gene loop, Ssu72 can recycle RNAPII by negatively regulating the phosphorylation of Ser5P at the termination region. TFIIB directly binds to RNAPII associated with the terminator activated by Ssu72. In this way, Ssu72 can stabilize the promoter–terminator gene loop with TFIIB. In addition, Ssu72 affects promoter directionality in relation to gene loops. The ncRNA made by RNAPII often starts with bidirectional promoters that synthesize mRNA and ncRNA in opposite directions.

Ssu72 participates in the dephosphorylation of the CTD by targeting Ser5P and Ser7P of the RNAPII CTD rather than Ser2P. Ssu72 is involved in the assembly of the PIC at the transcription start site [13]. It has been suggested that the Ssu72 phosphatase is associated with the CTD and other transcription factors. For example, Ssu72 depletion can disrupt transcriptional transitions in vitro and increase Ser5 phosphorylation towards the 3′-end of a gene [6,11]. During transcription initiation, Ssu72 can genetically and functionally interact with subunits of RNAPII (Figure 1A). The interplay between Ssu72 and RNAPII subunits including Rpb2 and Rpb4/7, which act as core RNAPII core machinery, can lead to PIC formation during transcription initiation. It has been shown that Ssu72 can physically stabilize the early elongation complex, indicating its functional association with magnesium-independent phosphatase activity [23,24]. The association between Ssu72 and TFIIB is highly conserved from yeast to humans. Ssu72 can promote the formation of TFIIB–TBP complexes, which are involved in PIC assembly by keeping TFIIB active. This functional interplay contributes to accurate transcriptional start site selection by RNAPII. Subsequently, Ssu72 has phosphatase activity at the Ser5 site, consistent with the notion that the Cys–X2–Cys–X6–Cys–X2–Cys zinc-binding motif, which is close to the N-terminus of Ssu72, and Cys residues in the motif are essential for the phosphatase activity of Ssu72 [6,13]. Ssu72 cooperates with diverse kinases and phosphatases involved in CTD phosphorylation [25]. In particular, it can interact with the CTD Ser5 kinase Kin28 to dephosphorylate CTD Ser5P and function in transcription initiation [8]. In addition, Ssu72 associates with the CTD phosphatase Fcp1 and Ctk1, which phosphorylates Ser2P in yeast, although some studies have revealed that Ssu72 has no Ser2P phosphatase activity because the Tyr1 residue does not fit into the tight β-turn of Ssu72 [8,26,27,28]. Ssu72 depletion can induce Fcp1 inactivation and early Ser2 phosphorylation by Ctk1, indicating that Ssu72 can tightly coordinate with various kinases and phosphatases during the transcription cycle [29].

Ssu72 dephosphorylates both Ser5P and Ser7P prior to termination. It plays an important role in regulating initiation–elongation and elongation–termination transitions (Figure 1A). After capping the 5’-end of nascent mRNA, Ssu72 can catalyze Ser5P in a Ser2-phosphorylation-dependent manner in concert with Ctk1, promoting the transition from initiation to elongation [30]. In addition, Ssu72 can function as a transcription elongation factor. Together with the Ser2P phosphatase Fcp1, Ssu72 can restore the hypophosphorylated form of RNAPII [6]. Recent studies have shown that Ess1, a proline isomerase, can interact with TFIIB and Ssu72 during transcription elongation and induce Ser5P–Pro6 CTD structural conformation during transcription termination [31]. It has been shown that this complicated interplay between Ssu72 and diverse kinases/phosphatases can affect the elongation–termination transition and facilitate the pause and release of RNAPII in response to a limited number of snoRNAs and transcripts of specific mRNAs, indicating that Ssu72 plays an essential role in the transcription termination process by facilitating RNAPII pause and release [8,31]. Because the role of Ssu72 in transcription termination is intimately involved in RNA 3′-end processing, this is elucidated in the section discussing 3′-end cleavage and polyadenylation by Ssu72 phosphatase. In later stages of transcription, Ssu72 can act as a factor controlling RNAPII ubiquitination and degradation. In the arrested elongation complex, RNAPII ubiquitination is dependent on the phosphorylation state of the CTD. RNAPII is susceptible to RNAPII ubiquitination and degradation in response to the phosphorylation of Ser5 sites of the CTD. Mutations of the region responsible for the Ser5 phosphatase activity of Ssu72 can lead to the increased Ser5P of the CTD, eventually inhibiting RNAPII degradation [32]. Accordingly, Ssu72 can promote the ubiquitination and degradation of RNAPII in response to CTD hyperphosphorylated Ser5.

In summary, the Ssu72 phosphatase can regulate each stage of the transcription process by catalyzing Ser5P and Ser7P in the CTD of RNAPII. A flexible interaction of Ssu72 with multiple factors results in dynamic structural plasticity in the CTD phosphorylation pattern. The failure of such interactions can cause inaccurate transcription cycles. Therefore, Ssu72 is indispensable for the establishment of the ‘CTD code’, by which the combination of CTD modifications generates numerous specific interactive surfaces [33,34].

### 2.2. 3′-end Cleavage and Polyadenylation by Ssu72 Phosphatase

#### 2.2.1. mRNA 3′-end Processing

Both 3′-end cleavage and polyadenylation require a cleavage and polyadenylation specificity factor complex (CPSF in humans; CPF in yeast) [35]. CPF can recognize signal peptides (AAUAAA) and induce the binding of cleavage stimulation factor (CstF) to form a protein complex at the 3’-end [36]. CPF can also stabilize poly (A) polymerase by binding to signal peptides [37]. Recently, the protein compositions of the CPF complex have been clearly identified. Both CPF and CPSF contain three subunits: a poly (A) polymerase, a nuclease, and a phosphatase subunit. In yeast, the poly (A) polymerase subunit comprises five proteins, four of which have human homologues: Yhh1 (CPF160), Yth1 (CPF30), Pfs2 (WDR33), Fip1 (hFip1), and poly (A) polymerase. The nuclease subunit comprises three proteins: Ysh1 (CPF73), Ydh1 (CPF100), and Mpe1 (RBBP6). The phosphatase subunit comprises six proteins: Pta1 (Symplekin), Ssu72, Swd2 (WDR82), Glc7 (PP1a/b), Pti1, and Ref2 [38,39].

Ssu72 is essential for 3′-end cleavage, poly (A) addition, and RNAPII termination [14]. Ssu72, as one of the components of CPF, can strongly interact with Pta1 and Pti1. It can also weakly interact with Ydh1p/Cft2p through the Rpb2 subunit of RNAPII. Thus, Ssu72 is also connected to CPF, TFIIB, and RNAPII [15]. Ssu72 cannot affect 3’-end processing without interacting with Pta1. Pta1 is a subunit of the CPF complex that functions in pre-mRNA cleavage, poly (A) addition, and transcription termination [40]. Pta1 and Ssu72 have strong genetic and physical interconnections, and their levels are closely related to each other’s in vivo [14]. The first 300 amino acids of the N terminus of Pta1 are important for interacting with Ssu72. It has been speculated that this region has an inhibitory effect on 3′-end processing. However, the interaction between this region and Ssu72 can block its inhibitory effect, leading to the cleavage and polyadenylation of Pta1 [40]. Symplekin can similarly bind to Ssu72. Symplekin, a metazoan homolog of Pta1, is thought to function as a scaffold protein [41]. It can interact with Ssu72 at residues 49–53, which constitute a flexible loop at the active site. This interaction can activate Ssu72′s phosphatase activity through either the stabilization of Ssu72′s structure or allosteric effects [42].

It has recently been shown that the peptidyl-prolyl isomerase Pin1 is related to the 3′ processing of Ssu72. The fission yeast Pin1 peptidyl-prolyl isomerase can catalyze the interconversion of *cis* and *trans* proline conformations [43]. It has previously been reported that Pin1 can increase Ssu72′s phosphatase activity for Ser7P and Ser5P [44]. As mentioned above, the CTD of the largest subunit of RNAPII contains a unique repeated heptad sequence of the consensus Tyr1–Ser2–Pro3–Thr4–Ser5–Pro6–Ser7 [45]. This phosphorylation profile comprises an informational code coordinating transcription and RNA processing. Recent studies have shown that CTD S2A, T4A, and S7A mutants can thrive with Pin1Δ (Pin1 null allele), whereas Y1F mutants and chimeric CTD mutants with half of the Pro3 or Pro6 residues replaced by alanine cannot thrive [43]. The expression of Pho1, Pho84, and Tgp1 is decreased by upstream long noncoding (lnc) RNA. These genes readily respond to changes in lncRNA 3′ processing/termination. Pin1Δ excessively inhibits the expression of the PHO gene and eliminates the inhibitory effect of CTD-S7A. Many coding RNAs are down- or upregulated in Pin1Δ cells, contributing to the inactivation of *cis*-proline-dependent Ssu72 CTD phosphatase activity [43,46]. Thus, Pin1 can positively influence 3′ processing/termination that is activated via Ssu72.

#### 2.2.2. Gene Looping

The RNAPII transcription cycle consists of distinct stages: the assembly of the PIC, initiation, elongation, termination, and reinitiation. When a gene is completely transcribed, RNAPII and newly synthesized RNA are released from the DNA template. The released RNAPII can revert to its basic hypophosphorylated state and participate in a new transcription process [47]. However, in many dynamically transcribing genes, promoter and terminator elements are juxtaposed to form an active structure called a ‘gene loop’ [16]. In the gene loop, RNAPII is transferred from the terminator to the promoter through a hand-off mechanism. The loop-like gene structure is formed by the interconnection between initiation and termination factors [48]. The gene loop, which is not a static structure, promotes RNAPII recycling and inhibits various transcriptions.

According to recent studies, gene looping is dependent upon transcription. It requires specific factors of transcription initiation and 3’-end processing complexes, including the Ssu72 CTD phosphatase [49]. In the gene loop, the interaction of Ssu72 with the PIC is involved in RNAPII recycling by dephosphorylating Ser5P at the termination region [14,50]. Ssu72 can stabilize promoter–terminator gene loops with TFIIB [51]. Hence, defects in Ssu72 and TFIIB can impair gene-loop formation. For example, the deletion of Ssu72 can lead to defects in TFIIB recruitment to promoters and eliminate TFIIB’s crosslinking to terminators [50]. Researchers have explained that TFIIB can directly bind to RNAPII associated with a terminator activated by Ssu72 (Figure 1B). Rpb4, a subunit of RNAPII, can promote the transfer of RNAPII from the terminator to the promoter for the reinitiation of transcription through TFIIB–Ssu72 interaction. In Δrpb4 mutant cells, gene looping is abolished and the association of TFIIB with the RNAPII complex is impaired. Ssu72 and Rpb4 may be included in the same complex during gene loop formation [16].

Ssu72 imposes promoter directionality in relation to gene loops. According to transcriptomics, only about 1.5% of a eukaryotic genome encodes proteins; the majority gives rise to non-coding RNAs, called ncRNAs. These ncRNAs made by RNAPII often start with bidirectional promoters that can synthesize mRNA and ncRNA in opposite directions (Figure 1B) [52]. The mutation of Ssu72 (Ssu72-2) can inhibit gene-loop formation over the FMP27 promoter. Ssu72-2 shows an increased density of RNAPII associated with the FMP27 promoter and promoter-associated antisense ncRNA. Ssu72 also exhibits a genetic interaction with the nuclear exosome component Rrp6, responsible for the degradation of many ncRNAs, especially CUTs (cryptic unstable transcripts). The disruption of gene-loop formation by the inactivation of Ssu72 leads to the production of abnormal ncRNAs that are stabilized in ΔRrp6 mutant cells. Like CUTs, Ssu72-restricted transcripts (SRTs) often progress in different directions from bidirectional promoters. Because gene-loop formation depends on Ssu72, which associates with both the promoter and the terminator, the inactivation of Ssu72 increases the synthesis of SRTs [53]. These findings indicate that Ssu72 can regulate the formation of gene loops and the transcriptional directionality of otherwise-bidirectional promoters.

## 3. Ssu72 in Chromosome Segregation, Telomere Regulation, and DNA Endoreplication

Most of the early studies on the Ssu72 phosphatase focused on its transcriptional functions in yeast, but recent reports have proposed that it is required as a chromosome-associated regulator, involved in chromosome segregation, telomere regulation, and hepatic chromosome polyploidization.

During mitosis, proper chromosome alignment and separation between sister chromatids are critical. This process occurs in a stepwise manner due to the involvement of the cohesin subunits [structural maintenance of chromosomes 1 (SMC1), SMC3, RAD21, and stromal antigen (SA)], which form a ring structure that topologically encircles DNA [54,55,56,57], and regulatory proteins such as Aurora B, cdk1, and plk1. In terms of the balance between the cohesion and segregation of sister chromatids, Ssu72 plays a novel role as a cohesin-binding phosphatase that can regulate sister chromatid cohesion and separation in early mitosis [1,58]. Ssu72 appears to directly interact with RAD21 and SA2 in vitro and in vivo, indicating that it regulates the cohesion and separation of chromosome arms by counteracting the phosphorylation of SA2. The mutational inactivation or deletion of Ssu72′s phosphatase activity can prevent the maintenance of sister chromatid cohesion by promoting early sister chromatid segregation, whereas Ssu72 overexpression appears to restore the resolution of sister chromatid arm cohesion. In addition, Ssu72 includes amino-acid sequences such as the polo-box-binding (PBB) motif recognized by Plk1 and a phosphorylation site for Aurora B. Another study supports the key role of Ssu72 in maintaining the integration of duplicated sister chromatids through the interaction between Aurora B and Ssu72 phosphatase [59]. Aurora B forms a complex with Ssu72 in vitro and in vivo, and promotes the phosphorylation of Ssu72 at Serine 19 during the G2 phase and early mitosis, contributing to the stability and activity of Ssu72. A Ssu72 phosphomimetic mutant phosphorylated by Aurora B indicates that Aurora B-mediated Ssu72 phosphorylation leads to the downregulation of Ssu72′s phosphatase activity. Furthermore, the mutation of Ssu72 S19 appears to be highly liable to trigger conformational change and reduced protein stability Ssu72, resulting in the ubiquitin-dependent degradation of Ssu72. Although Aurora B is required for efficient cohesin dissociation in prophase, it failed to directly phosphorylate cohesin subunits, suggesting the assistance of other factors or an indirect pathway [60]. Thus, the notion that Ssu72 acts as a direct target of Aurora B kinase-mediated phosphorylation furthers the understanding of the regulation of chromosome arm cohesion.

**Figure 2:**
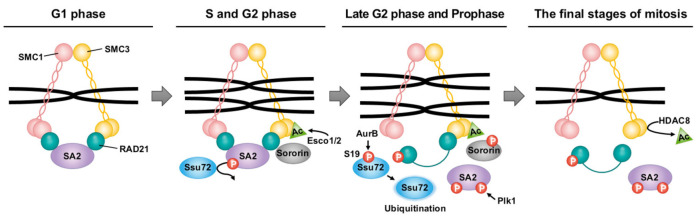
Schematic representation of Ssu72 phosphatase that regulates sister chromatid segregation during cell cycle. During the G1 phase, cohesin is regulated for the loading and unloading actions of NIPBL–MAU2 and PDS5–WAPL, respectively. In the subsequent step, Esco1/2 acetylates K105 and K106 of SMC3. Sororin reinforces the loading action via the recruitment of Pds5. In the late S phase, Ssu72 phosphatase can counteract the SA2 hyperphosphorylation associated with Cdk1 and Plk1, thus maintaining sister chromatid cohesion. However, the S19 residue with Ssu72 phosphatase activity is dephosphorylated through Aurora B-mediated phosphorylation in late G2 phase and prophase. Additionally, SA2 phosphorylation mediated by Plk1 can lead to the dissociation of cohesin from sister chromatids. Sororin is removed from cohesin by Aurora B. Finally, unresolved cohesins can retain their ring structure until the cleavage of the RAD21 subunit by separase. They can be reused in interphase after HDAC8 deacetylates the acetyl group of SMC3.

The comprehensive results suggest an attractive model in which Ssu72 regulates cohesion between sister chromatid arms and is phosphorylated by Aurora B in early mitosis, thereby contributing to the typical cell cycle regulation of cohesin. During the G1 phase, the loading action of NIPBL–MAU2 and the unloading action of PDS5–WAPL can affect the regulatory loading of cohesin to chromatids. First, cohesin is loaded into chromatin by the NIPBL–MAU2 heterodimer [55,61]. During the S and G2 phases, Esco1/2, a cohesin acetyltransferase, can acetylate the K105 and K106 residues of the N-terminal domain of SMC3 [62,63,64]. Sororin can prevent the unloading action by binding to Pds5, which displaces WAPL [65,66,67]. In the late S phase, the Ssu72 phosphatase can maintain sister chromatids’ cohesion by preventing the SA2 hyperphosphorylation mediated by Cdk1 and Plk1 (Figure 2) [59]. In prophase, Plk1 can remove cohesins from chromatin by phosphorylating the SA subunit. After being phosphorylated by Aurora B and Cdk1, sororin is released from cohesin [68,69].

In addition, the hyperphosphorylation of the Ser19 residue of Ssu72 by Aurora B can downregulate the phosphatase activity of Ssu72 (Figure 2) [58,59]. This post-translational modification of Ssu72 enables SA2 to be hyperphosphorylated by Plk1, leading to the dissociation of cohesins from chromosome arms [59]. By contrast, Sgo1 and PP2A reinforce the opposite reaction of cohesin dissociation by interfering with phosphorylation events through cohesin-associated proteins such as Aurora B and cdk1, allowing them to remain in the chromatin until anaphase [70]. The removal of RAD21 by separase activated by the anaphase-promoting complex cyclosome can induce the destruction of the cohesin ring structure [71]. The cohesin complex released during mitosis could be recycled in the interphase after cohesin deacetylase removes the acetyl group from SMC3 [72].

Ssu72 acts as an essential contributor in maintaining telomere homeostasis by controlling lagging-strand DNA synthesis in yeast and human cells [73]. Telomeres, located at the ends of eukaryotic chromosomes, protect chromosomes and maintain genomic stability [74,75]. Telomerases can compensate for the inevitable problems of telomeres such as replication fork collapse, with several DNA replication proteins and specific telomere components, ensuring appropriate telomere replication. Some studies provide evidence that the CTS complex (Cdc13–Stn1–Ten1 in *S. cerevisiae*) contributes to the regulation and termination of telomere replication [76]. The CTS complex not only promotes telomere lagging-strand DNA synthesis, but also terminates telomerase activity by counteracting it [77,78]. Recent results showing that Ssu72 regulates the recruitment of Stn1 to telomeres through Stn1 phosphorylation at Ser74 presents an additional explanation for the termination of telomere replication [73]. Ssu72Δ mutants show defective telomere replication and possess long 3′-ssDNA overhangs, indicating incomplete lagging-strand DNA synthesis. In human cells, SSU72 depletion leads to telomerase-dependent telomere elongation and increased telomere fragility. Furthermore, SSU72 depletion leads to the control of telomerase activation by the regulation of the recruitment of STN1 to telomeres. This supports the notion that Ssu72 regulates telomere lagging-strand synthesis by facilitating Stn1–polymerase α interaction, thereby terminating telomere replication and counteracting telomerase inhibition.

A recent study indicated that Ssu72 plays an essential role in maintaining hepatic chromosome endoreplication and liver function, which is vital for postnatal liver development from a physiological point of view. At birth, most of the hepatocytes, which perform a broad range of physiological functions for the regulation of liver metabolism, are diploids with single nuclei [79,80]. During postnatal liver development, the liver parenchyma undergoes gradual polyploidization, resulting in increased DNA content (teteraploidy, octoploidy, and so on) in each nucleus and a decrease in the number of nuclei per cell (mononucleate) [81,82]. Polyploidization-induced polyploid hepatocytes can produce daughter cells with numerous chromosomal abnormalities and uniparental chromosome sets. Such cells are susceptible to aneuploidy and/or apoptotic cell death in response to injury or stress signals [83,84]. Therefore, liver polyploidization is closely related to cell division control, including in DNA endoreplication, dissolving sister chromatid cohesion, and cytokinesis.

**Figure 3:**
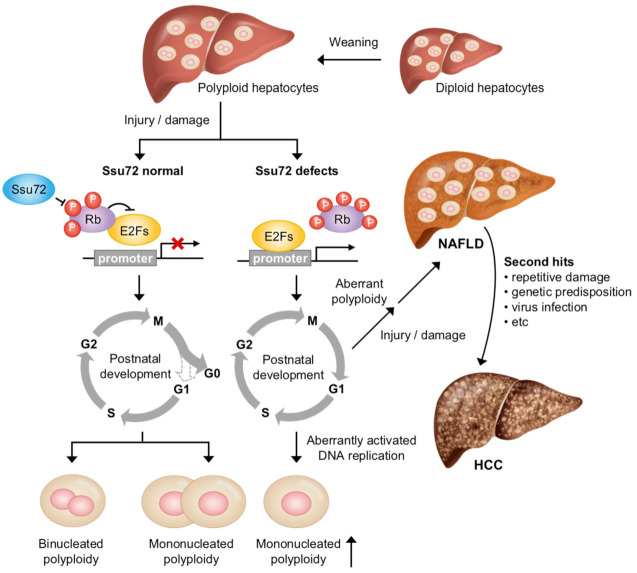
A physiopathological mechanism of Ssu72 regulating hepatic chromosome polyploidization and liver function during postnatal liver development. During postnatal development, Ssu72 plays an essential role in regulating hepatic chromosome polyploidization in a cell cycle-dependent manner. Depletion of Ssu72, which binds to and dephosphorylates Rb, contributes to the activation of E2F and then induces an aberrant DNA replication cycle by overriding the quiescence stage. Persistent elevation of the endoreplication process by Ssu72 depletion can facilitate the genesis of mononucleated polyploid hepatocytes, leading to extensive development of liver diseases such as NAFLD, fibrosis, and steatohepatitis. In addition, accumulation of liver damage such as from infection with HBV or HCV can affect the pathogenesis of HCC.

The mechanisms leading to physiological and pathological polyploid cells by liver polyploidization can be largely classified into DNA endoreplication, cytokinesis failure, and cell fusion, which is independent of the canonical cell cycle and division [85]. Notably, numerous factors contribute to the cell cycle-dependent process including DNA endoreplication and cytokinesis failure. For example, cell cycle-regulated factors such as Cdk1, pRb, E2F family, Ssu72, and survivin can affect liver polyploidization [86,87,88,89,90]. Recently, it has been demonstrated that the loss of Ssu72 phosphatase activity can increase polyploid hepatocytes through excessive endoreplication cycles, leading to pathogenic processes in the liver (Figure 3) [91]. When Ssu72, which binds to and dephosphorylates retinoblastoma protein (Rb), is depleted, the phosphorylation of Rb can activate E2Fs. Restriction point (R-point), which arrests cells in the late G1 phase of the cell cycle until cells receive extracellular signals to proceed to the S phase, is overridden by the aberrant activation of the DNA replication process. Eventually, the depletion of Ssu72 can result in mononucleated polyploid hepatocytes through excessive endoreplication cycles, inducing liver diseases including non-alcoholic fatty liver disease (NAFLD), fibrosis, and steatohepatitis (NASH) in a mouse model. This result highlights that Ssu72 might be a therapeutic target that can prevent the development of liver diseases.

## 4. Disease Implications of Loss of Ssu72′s Function

### 4.1. Hepatic Chromosome Polyploidy and Liver Function

The liver is an essential organ that has many functions in the body. It detoxifies various metabolites, synthesizes proteins, and produces biochemicals necessary for digestion and growth. The liver has several different types of cells, with hepatocytes accounting for 70% of all the liver’s parenchymal and non-parenchymal cells [92]. Liver function is primarily attributable to hepatocytes. The development of hepatocytes is marked by polyploidization, part of the developmental program, which increases the number of chromosomes per cell. Polyploidization occurs mainly during liver development. It can also occur in adults with increasing age or cellular stress (e.g., surgical resection, toxic exposure, or viral infection) [93,94]. This process mainly produces tetraploid and octoploid hepatocytes that are mononuclear (e.g., 4n or 8n) or binuclear (e.g., 2 × 2n or 2 × 4n). In liver development, most hepatocytes produce two diploid hepatocytes through a conventional cell cycle [86]. However, some hepatocytes might have a failure of cytokinesis, resulting in binuclear tetraploid hepatocytes. Those with failure of cytokinesis may develop into hepatocytes of varying ploidy, including diploidy, tetraploidy, octaploidy, and higher ploidy, by DNA endoreplication. Polyploid hepatocytes exist in mono- and binuclear forms. These polyploid hepatocytes ultimately account for about 90% of hepatocytes in adult mice and more than 50% in adult humans [95,96].

Polyploidy was first observed in plants more than a century ago, and it occurs in many eukaryotes under various circumstances. However, the mechanisms leading to liver polyploidization, how this process takes place in the liver parenchyma, and the functional consequences in liver proliferation remain largely unknown. One report has demonstrated a direct correlation between polyploidy and disease [97]. A decreased insulin level can abolish the generation of binucleated tetraploid hepatocytes, indicating that the impairment of insulin signaling can inhibit the generation of binucleated tetraploid hepatocytes. Indeed, a recent study supports the above notion that there is a direct association between abnormal hepatic polyploidy and the development of NAFLD [97]. In addition, a potential correlation of an increased nuclear size in hepatocytes with the development of NAFLD and NASH has been shown [98].

Recently, it has been shown that the deletion of Ssu72 specifically in hepatocytes (Albumin-Cre;Ssu72f/f, Ssu72Δhep) can cause several symptoms in the liver, including fat storage, necrosis, inflammatory cell infiltration, cytomegalic hepatocytes, and cytoplasmic vacuolization [97]. Furthermore, the deletion of Ssu72 in mice can activate a large population of hepatic stellate cells, known to be one of the main causes of liver fibrosis. Importantly, the deletion of Ssu72 can lead to the generation of aberrant hepatic polyploidy. Before weaning (three weeks of age), most hepatocytes isolated from WT and Ssu72Δhep mice were diploid (2c) and some were tetraploid (4c). After weaning (5–10 weeks of age), the WT hepatocytes had a high proportion of tetraploid and octaploid (8c) hepatocytes, with some diploid hepatocytes and an increased binucleation of hepatocytes. However, the Ssu72Δhep mice showed mostly octaploid hepatocytes, with almost no diploid hepatocytes, indicating a close correlation of aberrant chromosome ploidy with liver disease. Additionally, a deficiency of Ssu72 induced the hyperphosphorylation of the Rb protein [91]. It is well-known that the Rb phosphorylation status is closely related to the control of cell cycle progression and cell proliferation [99,100]. Rb is frequently inactivated in a number of pathogenic conditions, triggering the release of E2F1, which can activate downstream target genes required for the regulation of the cell cycle [101]. A recent report has shown that Ssu72 can regulate the transcriptional activity of E2F1 through interaction with Rb (Figure 3) [91]. Indeed, genomic analysis using hepatocytes isolated from Ssu72Δhep mice has shown that the transcription of genes related to cell cycle regulation is drastically increased, suggesting that the loss of Ssu72 can cause the aberrant activation of hepatocyte proliferation.

In summary, Ssu72 can maintain hepatic chromosome polyploidization and liver function. Therefore, the phosphatase activity of Ssu72 must be precisely regulated to maintain the normal polyploidy of hepatocytes. Previous studies suggest that regulating the expression or conversion of Ssu72 could be a means to elucidating physiological and pathological liver development.

### 4.2. Autoimmune Regulation by Homeostatic Balancing of T Cells

Autoimmune diseases are systemic or organ-specific diseases caused by the attack of the immune system on the body’s own tissues. Autoimmune diseases are associated with impaired control of the immune system [102]. Innate and adaptive immune systems are generally developed to protect the body. T lymphocytes contribute to adaptive immunity by regulating cellular immunity. CD4+ T helper (Th) cells that differentiate into effector subsets are important in regulating the immune response by secreting subset-specific cytokines. CD4+ Th cells are important for the tissue destruction observed in various inflammatory and autoimmune diseases such as multiple sclerosis, rheumatoid arthritis (RA), and inflammatory bowel disease (IBD) [103]. Defects in T cell populations and a failure to maintain self-tolerance can lead to autoimmune diseases [104].

RA is a symmetric, inflammatory, peripheral polyarthritis of unrevealed etiology. In RA, tendons and ligaments are stretched and joints are destroyed [105]. An imbalance of interleukin-17 (IL-17)-producing Th (Th17) cells/regulatory T cells (Treg) in the peripheral blood of RA patients contributes to RA development by increasing proinflammatory cytokines [106]. Signal transducer and activator of transcription 3 (STAT3) also induces inflammation and joint erosion in arthritic mouse models. The effect of STAT3 on the development of autoimmune diseases has been studied [107]. STAT3 activation can promote Th17 cell differentiation through the upregulation of IL-17a, thus aggravating RA development. Accordingly, STAT3 inhibition may be a potential target for RA therapy because this can block experimental autoimmune arthritis and regulate the balance between Th17 and Treg cells [108,109].

A recent study has provided important evidence that Ssu72 can effectively inhibit STAT3 activation [107]. The overexpression of Ssu72 can inhibit the production of proinflammatory cytokines and promote the expression of IL-4 and IL-10, which are related to T-cell mediated immunity. The overexpression of Ssu72 can also reduce the activation of STAT3-mediated signaling, including that of IL-17a and the IκB family members TBK1 and IKBKE. When Ssu72 expression is reduced, the activity of the IL-17a promoter and levels of p-STAT3 Tyr795 and Ser727 are increased, indicating that Ssu72 can directly regulate inflammatory immune responses through STAT3 signaling. Ssu72 directly interacts with STAT3. Interestingly, the introduction of Ssu72 to mice with type II collagen-induced arthritis (CIA) showed a therapeutic effect by reducing the levels of p-STAT3 Tyr705 and Ser727, subsequently decreasing the total IgE level and proinflammatory cytokines such as IL-1β, IL-17A, and tumor necrosis factor-α (TNF-α) [107]. Ssu72 can also dramatically reduce the infiltration of immune cells, joint destruction, and cartilage damage, indicating that it can reduce the severity of CIA by downregulating STAT3 activation and autoantibody production.

**Figure 4:**
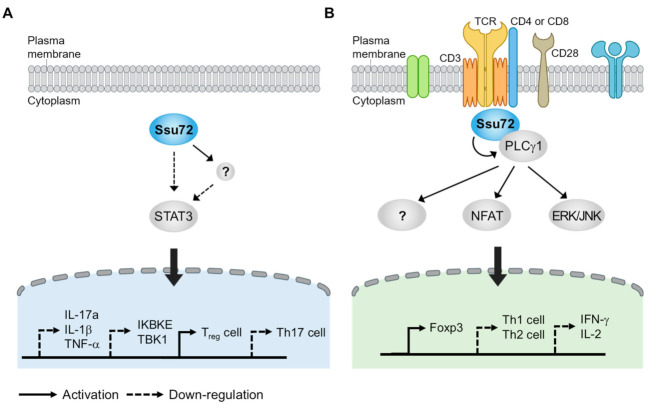
Ssu72 regulates autoimmune disease by controlling the homeostatic balance of T cell subsets. (**A**) Ssu72 regulates inflammatory responses by binding directly to STAT3. In the presence of Ssu72, Treg cell differentiation is increased while Th17 cell differentiation is decreased. mRNA levels of genes encoding proinflammatory cytokines, TBK1, and IKBKE are also downregulated. (**B**) Ssu72 is activated by various T cell receptor signaling molecules, including T cell receptor (TCR) and IL-2R. Activated Ssu72 can form a complex with PLCγ1, which is required for the development and function of Tregs. NFAT and ERK/JNK, downstream signaling molecules of PLCγ1, are then activated, promoting Foxp3^+^Treg induction. Ssu72 deficiency can prevent CD4 + T cell differentiation into Tregs at the peripheral region by inducing IL-2 and IFNγ, thus promoting CD4 + T cell activation.

Because the imbalance between Th17 and Treg cells contributes to the severity of autoimmune disease, regulating the mutual balance between these cell types is important for therapeutics [106,110]. Interestingly, murine splenocytes overexpressing Ssu72 show decreased differentiation into Th17 cells but increased differentiation into Treg cells [111], suggesting that Ssu72 can control the balance between Th17 and Treg differentiation. Moreover, the expression of p-STAT3 Tyr705 and Ser727 and the differentiation into Th17 cells are markedly reduced when splenocytes isolated from CIA mice stimulated with IL-6 are treated with purified Ssu72 protein [111]. These results again suggest that Ssu72 can reduce Th17 cell differentiation and inflammatory responses by inhibiting STAT3 activation. It has been widely reported that phosphatase-mediated signaling cascades are largely associated with the development of autoimmune diseases. Thus, targeting phosphatase might be a novel strategy for treating autoimmune diseases. For example, modulating receptor protein tyrosine phosphatase alpha activity can improve arthritis in arthritic experimental models such as CIA and K/BxN serum transfer mouse models [112,113]. Furthermore, other phosphatases involved in the regulation of STAT3 activation such as DUSP2 and CIP2A can improve treatment efficacy by reducing Th17 cell differentiation [114,115]. Although how Ssu72 affects STAT3 phosphorylation remains unclear, this evidence indicates that Ssu72 can attenuate STAT3 activation and Th17 cell differentiation. Thus, it is possible to treat autoimmune diseases by regulating Ssu72 phosphatase activity (Figure 4A).

IBD is an inflammatory disease that occurs in the colon and small intestine. Crohn’s disease and ulcerative colitis are typical examples of IBD [116]. Based on our recent observation, we found that Ssu72 was strongly activated by anti-CD3/28. Interestingly, naïve CD4+ T cells, which are activated by anti-CD3/28 in combination with IL-2 and TGF- β, frequently recruit Ssu72 to the cytoplasmic side of the cell membrane, indicating that Ssu72 might act as a receptor-signal-responsive phosphatase in T cells. In addition, Ssu72 that is expressed on Foxp3+ regulatory T cells is downregulated in active IBD [117]. Foxp3+ Tregs are generally produced in the thymus (tTreg), although they can also be produced in peripheral regions (pTreg) or induced through cell culture (iTreg) in the presence of transforming growth factor-β (TGF-β) [118]. pTregs against non-self antigens are required for the maintenance of the tolerance of mucosal surfaces including in the intestines and lungs [119]. Our recent observation revealed that Ssu72 depletion resulted in a significant decrease in the proportion of pTregs in vivo and inhibited the differentiation of naïve CD4+ T cells into iTregs in vitro (Figure 4B). Ssu72 seems regulate the PLCγ1 downstream signaling and subsequently Foxp3 expression. Furthermore, we found that Ssu72 was downregulated in ulcerative colitis (active phase) and Crohn’s disease (active phase) [117]. Taken together, these findings suggest that Ssu72 is required for Treg differentiation and important for mucosal homeostasis.

## 5. Therapeutic Perspectives

The coordinated interplay of protein kinases and phosphatases orchestrates phosphorylation homeostasis. However, the relationship between physiological mechanisms and pathogenesis regarding phosphatases is less characterized than that regarding protein kinases [120]. Recent studies have suggested that protein phosphatases such as PTEN, PP2A, and Ssu72 play essential roles and are therapeutic targets in many diseases [121,122]. Notably, the Ssu72 phosphatase, belonging to the subfamily of DUSPs, has a wide range of effects on aspects ranging from transcription biogenesis to human pathophysiology. We have discussed how newly discovered physiological functions of Ssu72, a phosphatase, could influence the pathogenesis of diverse diseases including NAFLD and autoimmune diseases. Based on this, we can propose potential strategies for developing therapeutics by regulating Ssu72.

Ssu72 is involved in the transcription cycle and RNA processing through the dephosphorylation of Ser5P and Ser7P in the CTD of RNAPII. Ssu72 intervenes at different stages of the transcription process by interacting with RNAPII subunits including Rpb2, TFIIB, and other mediators. Additionally, Ssu72 along with CPF components can regulate the 3′-end processing of nascent mRNAs and gene looping, indicating that Ssu72 is indispensable for transcription biogenesis. Ssu72 depletion can lead to abnormal RNAPII pausing and elongation defects, thereby decreasing the efficiency of elongation during RNAPII-mediated gene expression. Recent studies have shown that Ssu72 phosphatase could be a target for treating viral infectious diseases. For example, Ssu72, which mediates transcription termination and gene looping for eukaryotic genes, is required in HIV-1 Tat transactivation. HIV-1 Tat enables Ssu72 to stimulate viral gene expression by direct interaction [123]. In addition, Ssu72 is associated with transcriptional read-through (TRT), an RNA molecule formed via the splicing of exons, and influenza polymerase–CTD binding, indicating the possibility of regulating the expression of viral genes and allowing Ssu72 to function for efficient viral transcription and the development of targeted antiviral drugs [124]. Another study has suggested that, when Ssu72 forms a complex with symplekin, which activates Ssu72, the active site of Ssu72 only binds to a CTD peptide with a SerP5–Pro6 peptide bond in cis configuration [9]. Together, these results suggest that the active site of Ssu72 could be a target of phosphatase activity for polyadenylation during transcription.

Ssu72 not only physiologically functions as a cohesin-binding phosphatase that modulates sister chromatid separation in early mitosis, but also plays a role as a gatekeeper to maintain hepatic chromosome integrity and monitor liver function. Notably, the mutation or loss of Ssu72 appears to inhibit sister chromatid cohesion, whereas the overexpression of an Aurora B-mediated Ser19 mutant of Ssu72 restores chromosome arm cohesion, suggesting that Ssu72 might play a key role in regulating sister chromatid cohesion during early mitosis [58,59]. Additionally, the depletion of Ssu72 contributes to hepatic disorders, including necrosis, steatohepatitis, and fibrosis, via aberrant hepatic chromosome polyploidization [91]. The downregulation of Ssu72 is highly conducive to the development of NAFLD/NASH-associated hepatocellular carcinoma (HCC). Therefore, targeting Ssu72 could be a potential strategy for the treatment of both NASH and HCC.

As mentioned previously, Ssu72 could be a target for autoimmune diseases. Strikingly, Ssu72 is associated with the pathogenesis of RA through STAT3 activation and Th17 cell differentiation. The overexpression of Ssu72 can attenuate STAT3 activation and Th17 cell responses in vitro [111]. Additionally, Ssu72 can substantially alleviate RA progression by decreasing inflammatory responses and switching the Th17/Treg balance, indicating that Ssu72 could have therapeutic effects on RA. Our recent observation also suggested that Ssu72 could positively regulate TCR and TGF-β signaling and promote the homeostasis of mucosal tolerance. A deficiency of Ssu72 in T cells can induce an inflammatory response in mucosal tissues including those in the intestines, subsequently leading to susceptibility to the development of IBD [117]. Furthermore, a recent study has reported that Ssu72 in tissue-resident macrophages can regulate the development and maturation of alveolar macrophages (AM) and allergic airway inflammation through GM-CSFR signaling [125]. Ssu72-deficient AMs resulted in the dysregulation of mitochondrial respiration, cell death, and the cell cycle after stimulation with GM-CSF, contributing to the immunological roles of Ssu72 in a tissue-specific manner.

In summary, due to its transcriptional and physiological functions, Ssu72 plays pivotal roles as a therapeutic target and a biomarker. Recent mechanistic insights into the Ssu72 phosphatase encompass a spectrum of diverse disorders with Ssu72-depleted mouse models. However, the physiological mechanisms of Ssu72 that lead to the development of diseases remain elusive. Further studies are needed to elucidate the elaborate correlations between the physiological functions of Ssu72 and pathological states.

## Data Availability

Not applicable.
